# Construction of diagnostic and prognostic models for premature coronary artery disease based on multiple machine algorithms

**DOI:** 10.3389/fcvm.2025.1611709

**Published:** 2025-11-17

**Authors:** Yu-Chan He, Ye Li, Xiu-Jin Qin, Zhong-Hai Bi, Si-Yao Li, Yan-Li Liu, Liu Miao

**Affiliations:** 1 Department of Cardiology, Liuzhou People’s Hospital, Affiliated of Guangxi Medical University, Liuzhou, Guangxi, China; 2 The Key Laboratory of Coronary Atherosclerotic Disease Prevention and Treatment of Liuzhou, Liuzhou People's Hospital, Liuzhou, Guangxi, China

**Keywords:** premature coronary artery disease, pan-Immune-Inflammation value, triglyceride-glucose index, machine learning, diagnostic model, prognostic model, survival analysis

## Abstract

**Objective:**

To evaluate the diagnostic and prognostic predictive value of the pan-immune-inflammation value (PIV) and triglyceride-glucose (TyG) index in premature coronary artery disease (PCAD).

**Methods:**

This study analyzed data from 26,883 patients admitted with chest pain at Liuzhou People's Hospital (January 2014 to December 2020), with 5,653 patients included after screening. Multiple machine learning algorithms, including Gradient Boosting Machine (GBM), Extreme Gradient Boosting (XGBoost), Support Vector Machine (SVM), Lasso regression, Random Forest (RF), and logistic regression, were applied to identify PCAD-related variables, which were integrated into a decision tree model. Propensity score matching (PSM) ensured cohort comparability. The Mime1 package facilitated ensemble feature selection and visualization, while optimal PIV and TyG cutoff values were determined via Receiver Operating Characteristic (ROC) analysis for 36-month survival subgroup analysis.

**Results:**

Logistic regression identified PIV [odds ratio [OR] 2.651, 95% CI [to be specified], *P* < 0.001] and TyG [OR 1.003, 95% CI (to be specified), *P* < 0.001] as PCAD risk factors. The decision tree model, incorporating PIV, TyG, and white blood cell count (WBC), achieved an accuracy of 0.88 and an area under the ROC curve (AUC) of 0.86 for PCAD diagnosis. Survival analysis over 36 months revealed that low PIV and TyG levels were associated with reduced all-cause mortality, whereas elevated levels correlated with poorer prognosis (*P* < 0.001), with TyG showing a pronounced effect.

**Conclusion:**

The combined evaluation of PIV, TyG, and WBC offers robust diagnostic and prognostic value for PCAD, with elevated PIV and TyG levels indicating a poor prognosis, underscoring their potential as clinical biomarkers.

## 1 Introduction

Coronary artery disease (CAD) remains the leading cause of morbidity and mortality worldwide, despite advances in prevention and treatment strategie (1, 2). Premature coronary artery disease (PCAD), defined as CAD occurring in men younger than 55 years and women younger than 65 years, represents a distinct clinical entity. Compared with CAD in older individuals, PCAD often presents more acutely, carries a higher lifetime risk of recurrent events, and imposes a substantial socioeconomic burden (3, 4).

Traditional risk factors such as smoking, hypertension, dyslipidemia, and diabetes contribute to PCAD development, but emerging evidence highlights the role of inflammation and metabolic dysregulation (5–7). Atherosclerosis is increasingly recognized as a chronic inflammatory condition driven by endothelial injury, lipid accumulation, and immune responses (8, 9). Consequently, inflammatory and metabolic biomarkers have attracted interest for risk stratification in younger CAD populations.

The pan-immune-inflammation value (PIV), derived from neutrophil, platelet, monocyte, and lymphocyte counts, has been validated as a prognostic marker in cancer and cardiovascular disease (10–12). Similarly, the triglyceride-glucose (TyG) index, an established surrogate of insulin resistance, has been shown to predict cardiovascular events across diverse populations (13, 14). Both markers are readily obtainable from routine laboratory tests, making them practical for clinical application.

However, limited studies have examined the combined prognostic value of PIV and TyG in patients with PCAD. Given the unique metabolic and inflammatory profile of younger patients, evaluating these indices may provide insights into risk stratification and prognosis.

## 2 Method

### 2.1 Research design

**Figure 1:**
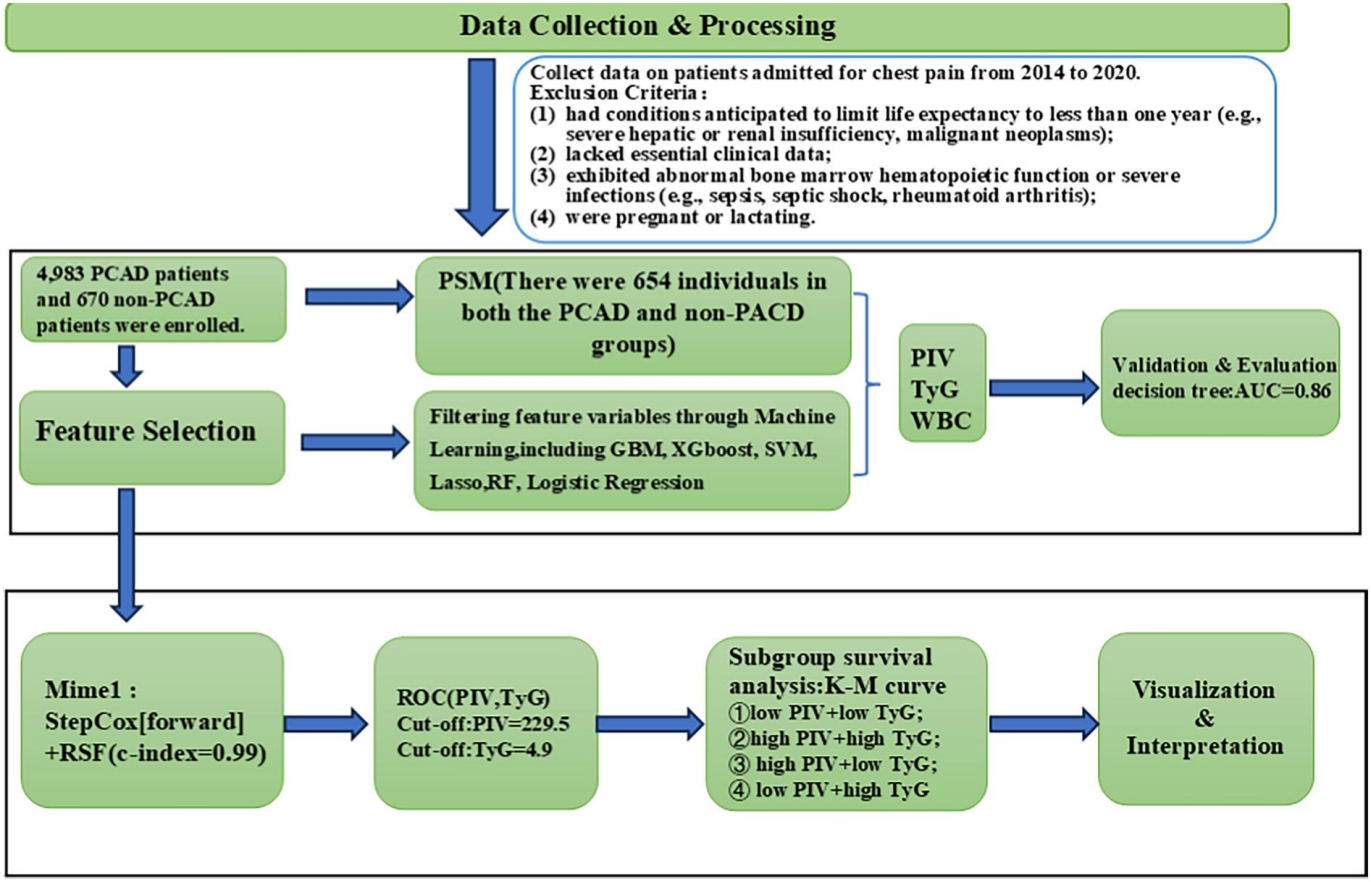
Model development and validation flowchart. PCAD, premature coronary artery disease; PIV, pan-immune inflammatory value; TyG, triglyceride-glucose index; WBC, white blood cell count; PSM, propensity score matching; GBM, gradient boosting machine; XGboost, extreme gradient boosting; SVM, support vector machine; RF, random forest; RSF, random survival forest; ROC, receiver operating characteristic.

This study was designed to investigate risk factors linked to premature coronary artery disease (PCAD) and their prognostic importance through the application of multiple machine learning approaches. The analysis utilized a comprehensive dataset of clinical records to provide a foundation for early screening and primary prevention strategies. A schematic overview of the study workflow is shown in Figure 1, depicting the sequential process of data acquisition, variable selection, model development, and validation.

### 2.2 Study population

This study evaluated clinical data from 26,883 patients consecutively admitted to Liuzhou People's Hospital from January 2014 to December 2020. Patients were eligible for inclusion if they met the following criteria: (1) presented with chest pain and underwent coronary angiography; (2) were males younger than 55 years or females younger than 65 years; and (3) were diagnosed with coronary artery disease (CAD) based on ≥50% stenosis in at least one major coronary artery (left main, left anterior descending, circumflex, or right coronary artery). Patients were classified into premature CAD (PCAD) or non-PCAD groups according to coronary angiographic results. Patients were excluded if they met any of the following criteria: (1) had conditions anticipated to limit life expectancy to less than one year (e.g., severe hepatic or renal insufficiency, malignant neoplasms); (2) lacked essential clinical data; (3) exhibited abnormal bone marrow hematopoietic function or severe infections (e.g., sepsis, septic shock, rheumatoid arthritis); or (4) were pregnant or lactating. Ultimately, 4,983 PCAD patients and 670 non-PCAD patients were enrolled. Patients were followed up regularly at 3, 6, 9, and 12 months after enrollment through telephone follow-up, readmission follow-up, and outpatient follow-up. The total follow-up period was 36 months. The observational endpoint was defined as all-cause mortality, including cardiovascular death (death due to myocardial infarction, heart failure, arrhythmias, or other cardiovascular causes), with follow-up concluding at the time of death. All-cause mortality served as the measure of prognosis.

### 2.3 Data collection

A comprehensive dataset was constructed from patient medical records, encompassing baseline characteristics including sex, age, body mass index (BMI), blood pressure, history of smoking and alcohol consumption, and fasting blood glucose concentrations. Furthermore, the initial blood sample collected upon admission was assessed for a range of biomarkers and indices, including white blood cell count (WBC), red blood cell count (RBC), hemoglobin (Hb), platelet (PLT), hematocrit (Hct), eosinophil count, lymphocyte, monocyte, basophil, neutrophil, total cholesterol (TC), triglycerides (TG), low-density lipoprotein cholesterol (LDL-C), high-density lipoprotein cholesterol (HDL-C), creatinine (Scr), urea nitrogen (BUN), homocysteine (Hcy), aspartate aminotransferase (AST), alanine aminotransferase (ALT), waistline(WC), glycated hemoglobin (HbA1), triglyceride-to-HDL ratio (THR),triglyceride-glucose index (TyG), PIV, Charlson Comorbidity Index (CCI), Body Roundness Index (BRI), Visceral Adiposity Index (VAI), and Castelli Risk Indices 1 and 2 (CRI-1, CRI-2). The TyG index was calculated as ln[fasting triglycerides (mg/dl) × fasting glucose (mg/dl)/2], and the PIV as (neutrophil count × platelet count × monocyte count)/lymphocyte count.

### 2.4 Statistical analysis

All statistical analyses were performed using R software. Continuous variables are presented as means ± standard deviations, with between-group comparisons conducted using analysis of variance (ANOVA). Categorical variables are reported as counts and percentages, with group differences evaluated using the chi-square test or Fisher's exact test, depending on applicability. A *P*-value < 0.05 was deemed statistically significant. Propensity score matching (PSM) was employed to adjust for potential confounders and ensure comparability between the premature coronary artery disease (PCAD) and non-PCAD cohorts. Variables predictive of PCAD diagnosis were determined using a range of machine learning methods, including gradient boosting machine (GBM), extreme gradient boosting (XGBoost), support vector machine (SVM), Lasso regression, random forest, and logistic regression. A decision tree model was subsequently developed to formulate a diagnostic algorithm for PCAD. For patients fulfilling the PCAD diagnostic criteria, receiver operating characteristic (ROC) curves were used to establish optimal cutoff values for the PIV and the TyG index. These cutoff values were used to stratify the population into four subgroups: low PIV, high PIV, low TyG, and high TyG. Kaplan–Meier survival curves were constructed to assess prognostic differences among these subgroups.

## 3 Result

### 3.1 Patient characteristics

A total of 26,683 patients admitted with chest pain were enrolled. Of these, 21,030 patients were excluded for not meeting the inclusion criteria. Consequently, 5,653 patients were included in the final analysis.

### 3.2 Initial search for characteristic variables

The study comprised 5,653 participants, including 4,983 patients with PCAD and 670 with non-PCAD. Following PSM, the cohorts were balanced, yielding 654 participants per group. Before PSM, significant differences emerged between the PCAD and non-PCAD groups for numerous variables (all *P* < 0.001), including sex, white WBC, RBC, Hb, Hct, eosinophil count, lymphocyte count, PLT, CRP, HDL-C, CRI-1, CRI-2, PIV, CCI, TG, LDL-C, BRI and THR. After PSM, differences remained significant for a subset of variables (all *P* < 0.001), WBC, RBC, Hb, neutrophil count, PLT, CRP, TyG, PIV, and CCI. Notably, the TyG index, PIV, and WBC consistently differed between the PCAD and non-PCAD groups both before and after PSM. These results indicate that these variables may contribute to PCAD (Table 1).

**Table 1:** General characteristics of patients before and after PSM.

Parameter	Before			After		
	Non-PCAD (*n* = 4,983)	PCAD (*n* = 670)	*P* value	Non-PCAD (*n* = 654)	PCAD (*n* = 654)	*P* value
Age	40.29 (12.15)	39.75 (11.81)	0.277	40.06 (11.81)	39.79 (11.79)	0.67
Gender = Male	2,127 (42.7)	338 (50.4)	<0.001	337 (51.5)	328 (50.2)	0.658
WBC	4.74 (0.49)	4.52 (0.40)	<0.001	4.78 (0.51)	4.52 (0.40)	<0.001
RBC	4.66 (0.49)	4.50 (0.56)	<0.001	4.65 (0.53)	4.50 (0.56)	<0.001
Hb	14.05 (1.54)	13.67 (1.64)	<0.001	14.07 (1.57)	13.68 (1.63)	<0.001
Hct	41.48 (4.27)	40.53 (4.66)	<0.001	41.52 (4.43)	40.56 (4.64)	<0.001
Eosinophil	0.18 (0.17)	0.22 (0.23)	<0.001	0.21 (0.18)	0.22 (0.23)	0.303
Lymphocyte	2.01 (0.99)	1.86 (1.63)	0.001	2.03 (1.34)	1.86 (1.65)	0.042
Monocyte	0.50 (0.24)	0.55 (0.28)	<0.001	0.53 (0.23)	0.54 (0.28)	0.532
Basophil	0.04 (0.06)	0.04 (0.07)	0.116	0.04 (0.06)	0.04 (0.08)	0.967
Neutrophil	2.84 (0.30)	2.94 (0.26)	<0.001	2.87 (0.31)	2.94 (0.26)	<0.001
PLT	254.94 (67.51)	237.50 (71.79)	<0.001	253.24 (73.67)	237.52 (71.74)	<0.001
Alt	26.66 (20.57)	28.32 (75.34)	0.213	26.87 (19.19)	28.21 (76.13)	0.662
Ast	25.39 (18.82)	24.83 (15.85)	0.463	24.89 (14.71)	24.81 (15.98)	0.923
BUN	4.34 (1.63)	4.34 (1.58)	0.918	4.32 (1.41)	4.34 (1.59)	0.798
Scr	103.47 (18.80)	104.16 (19.75)	0.379	103.09 (18.15)	104.00 (19.83)	0.387
Hcy	0.78 (2.81)	1.00 (3.76)	0.069	0.88 (2.89)	1.02 (3.80)	0.438
CRP	0.23 (0.61)	0.39 (0.90)	<0.001	0.25 (0.58)	0.40 (0.91)	<0.001
HDL	1.38 (0.41)	1.30 (0.39)	<0.001	1.29 (0.36)	1.30 (0.39)	0.39
LDL	2.96 (0.93)	3.06 (0.91)	0.008	3.10 (0.94)	3.06 (0.92)	0.412
TC	5.01 (1.05)	5.03 (1.08)	0.611	5.09 (1.04)	5.03 (1.08)	0.338
TG	1.36 (0.80)	1.46 (0.84)	0.003	1.50 (0.86)	1.46 (0.84)	0.361
Glu	104.07 (30.41)	104.01 (31.57)	0.957	104.18 (29.90)	103.99 (31.87)	0.912
HbA1c	5.55 (0.98)	5.56 (0.99)	0.933	5.57 (0.98)	5.56 (1.00)	0.858
SBP	118.84 (15.70)	119.54 (16.18)	0.279	119.19 (15.60)	119.57 (16.18)	0.664
DBP	70.98 (11.55)	70.89 (11.65)	0.856	72.07 (11.85)	70.98 (11.74)	0.093
BMI	28.87 (6.81)	28.90 (6.92)	0.908	29.00 (6.61)	28.95 (6.94)	0.897
WC	97.76 (16.28)	97.60 (16.78)	0.821	98.45 (16.05)	97.70 (16.84)	0.413
Smoke = Yes (%)	2,396 (48.1)	324 (48.4)	0.926	322 (49.2)	316 (48.3)	0.782
Drink = Yes (%)	806 (16.2)	110 (16.4)	0.917	118 (18.0)	105 (16.1)	0.378
CRI1	3.90 (1.27)	4.20 (1.56)	<0.001	4.23 (1.38)	4.19 (1.54)	0.555
CRI2	2.33 (1.00)	2.58 (1.16)	<0.001	2.60 (1.09)	2.57 (1.14)	0.588
THR	1.15 (0.92)	1.33 (1.16)	<0.001	1.35 (1.06)	1.33 (1.14)	0.66
TyG	4.08 (0.62)	4.17 (0.58)	0.001	4.09 (0.63)	4.17 (0.58)	0.004
BRI	5.40 (2.22)	5.15 (2.33)	0.007	5.15 (2.04)	5.16 (2.33)	0.928
PIV	176.11 (111.33)	209.69 (149.0)	<0.001	201.37 (158.93)	208.68 (149.78)	0.007
CCI	0.82 (1.27)	2.58 (1.98)	<0.001	2.45 (1.93)	2.47 (1.85)	0.826

PCAD, premature coronary artery disease; WBC, white blood cell count; RBC, red blood cell count; Hb, hemoglobin; Hct, hematocrit; PLT, platelets; ALT, alanine aminotransferase; AST, aspartate aminotransferase; BUN, urea nitrogen; Scr, creatinine; Hcy, homocysteine; CRP, c-reactive protein; HDL, high-density lipoprotein cholesterol; LDL, low-density lipoprotein cholesterol; TC, total cholesterol; TG, triglycerides; Glu, blood glucose; HbA1c, glycated hemoglobin; SBP, systolic blood pressure; DBP, diastolic blood pressure; BMI, body mass index; WC, waistline; CRI1and CRI2, castelli risk index 1 and 2; THR, triglyceride-to-HDL ratio; TyG, triglyceride-glucose index; BRI, body roundness index; PIV, pan-immunoinflammatory value; CCI, Charleston comorbidity index.

### 3.3 Filtering feature variables through machine learning

Machine learning analyses were performed on the 37 previously described feature variables using R.

**Figure 2:**
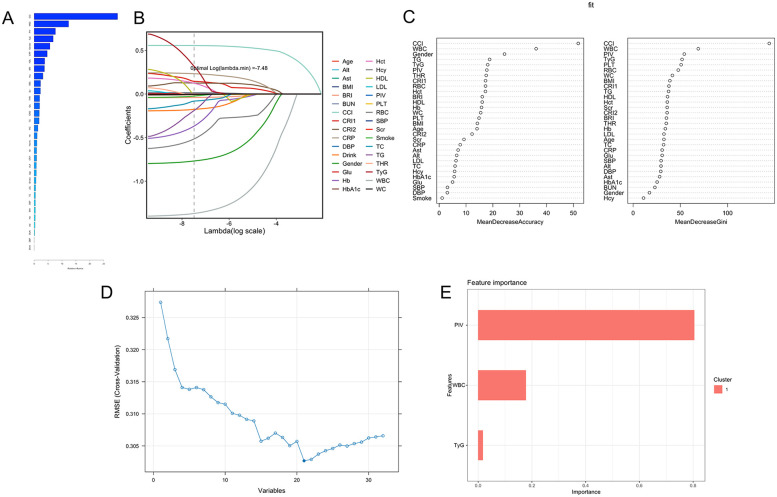
Multiple machine learning results. **(A)** Gradient Boosting Machine (GBM): The top five feature variables based on relative importance ranking are CCI, WBC, Gender, PIV, and TyG. **(B)** Lasso regression: at the optimal lambda value (lambda = −7.48), 32 variables were retained, including TyG, PIV, and WBC. **(C)** Random forest: based on Gini importance evaluation, the five most important features are CCI, WBC, PIV, TyG, and PLT. **(D)** Support vector machines (SVM): The model achieved the lowest Root Mean Square Error (RMSE) of 0.3027, indicating optimal performance, with the top five contributing variables being CCI, WBC, RBC, PIV, and TyG. **(E)** Extreme Gradient boosting (XGBoost): Based on the feature importance scores, the variables are ranked as follows: PIV (0.80), WBC (0.18), and TyG (0.02).

Gradient Boosting Machine (GBM): GBM, an ensemble learning algorithm, sequentially integrates multiple weak learners to reduce prediction error. Using GBM, variable importance was assessed, identifying the top five variables as the CCI, WBC, sex, PIV, and TyG (Figure 2A).

Lasso Regression: Lasso regression was applied for feature selection and model regularization. With an optimal lambda value of −7.48, key variables were identified and ranked in descending order of importance: age, alanine aminotransferase (ALT), aspartate aminotransferase (AST), BMI, BRI, blood urea nitrogen (BUN), CCI, CRI-1, CRI-2, CRP, diastolic blood pressure (DBP), alcohol consumption, sex, glucose (Glu), Hb, glycated hemoglobin (HbA1c), Hct, homocysteine (Hcy), HDL-C, LDL-C, PIV, PLT, RBC, systolic blood pressure (SBP), serum creatinine (Scr), smoking status, TC, TG, THR, TyG, WBC, and waist circumference (WC) (Figure 2B).

Random Forest (RF): The RF algorithm improves prediction accuracy, generalization, and resistance to overfitting through the aggregation of multiple decision trees (via averaging for regression or majority voting for classification). Risk factors for PCAD were evaluated for importance, with higher values denoting greater significance. The top five variables identified were the CCI, WBC, PIV, TyG, and PLT (Figure 2C).

Support Vector Machine (SVM): The SVM classification algorithm was applied to classify the data. Iterative model training and sequential removal of less significant variables enabled the identification of the most predictive features. The model was optimized by minimizing root mean squared error (RMSE), resulting in the selection of 21 variables, with the top five being the CCI, WBC, RBC, PIV, and TyG (Figure 2D).

Logistic Regression: Logistic regression was employed to model the binary classification of PCAD diagnosis (PCAD = 1, non-PCAD = 0). A stepwise method was used to select variables, yielding a final model with nine key predictors: RBC, WBC, PLT, PIV, TyG, sex, Hb, Hct and CRP (Table 2).

**Table 2:** Logistic regression.

Parameter	SMD	OR	*P* value	Parameter	SMD	OR	*P* value
Age	1.262	1.002	0.665	Hcy	0.014	1.012	0.395
Gender	0.005	0.419	<0.001	CRP	0.057	1.388	<0.001
WBC	0.109	0.254	<0.001	HDL	0.332	1.499	0.222
RBC	0.103	0.403	<0.001	LDL	0.151	1.074	0.634
Hb	0.157	0.513	<0.001	TC	0.159	0.814	0.196
Hct	0.115	1.267	<0.001	TG	0.332	0.483	0.029
Eosinophil	0.048	1.590	0.133	Glu	0.002	0.993	0.015
Lymphocyte	0.012	5.275	0.444	HbA1c	0.060	0.994	0.931
Monocyte	0.175	3.871	0.213	SBP	0.003	1.006	0.046
Basophil	0.094	4.244	0.304	DBP	0.004	0.992	0.101
Neutrophil	0.103	6.816	0.429	BMI	0.0,170	1.019	0.261
PLT	0.001	0.993	<0.001	WC	0.007	0.988	0.111
Alt	0.001	0.999	0.806	Smoke	0.093	0.960	0.661
Ast	0.003	0.997	0.423	Drink	0.125	0.959	0.742
BUN	0.032	1.017	0.591	CRI1	0.179	1.278	0.172
Scr	0.032	1.002	0.417	CRI2	0.172	1.046	0.793
TyG	0.287	2.6511	<0.001	BRI	0.021	0.952	0.019
PIV	0.004	1.0037	<0.001	THR	0.236	1.162	0.523

WBC, white blood cell count; RBC, red blood cell count; Hb, hemoglobin; Hct, hematocrit; PLT, platelets; ALT, alanine aminotransferase; AST, aspartate aminotransferase; BUN, urea nitrogen; Scr, creatinine; TyG, triglyceride-glucose index; PIV, pan-immunoinflammatory value; Hcy, homocysteine; CRP, c-reactive protein; HDL, high-density lipoprotein cholesterol; LDL, low-density lipoprotein cholesterol; TC, total cholesterol; TG, triglycerides; Glu, blood glucose; HbA1c, glycated hemoglobin; SBP, systolic blood pressure; DBP, diastolic blood pressure; BMI, body mass index; WC, waistline; CRI1and CRI2, castelli risk index 1 and 2, BRI, body roundness index; THR, triglyceride-to-HDL ratio; SMD, standardized mean difference; OR, odds ratio.

Extreme Gradient Boosting (XGBoost): The XGBoost algorithm was applied to evaluate the relative importance of feature variables, identifying the top three as the PIV, TyG, and WBC (Figure 2E).

These machine learning results are presented in Figure 2. Notably, the consistent prominence of the feature variables—specifically the TyG, PIV and WBC—corresponds with their observed differences between the PCAD and non-PCAD groups both before and after PSM.

### 3.4 Diagnostic prediction model

**Figure 3:**
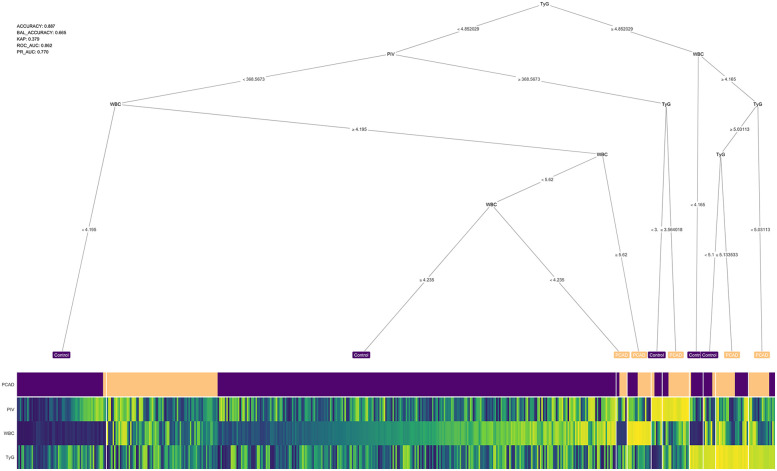
Decision tree. The accuracy of the model is 0.88 and the confidence level is 0.86. WBC, white blood cells; TyG, triglyceride-glucose index; PIV, pan-immunoinflammatory value.

A decision tree model was constructed to predict PCAD based on key feature variables namely, the PIV, WBC and TyG identified through multiple machine learning algorithms. The decision tree applies conditional rules to classify PCAD, outlined as follows: (1) If TyG ≥ 4.85 and WBC > 4.165 × 10⁹ /L: (a) TyG > 5.13 indicates PCAD; (b) 4.85 ≤ TyG ≤ 5.03 with WBC > 4.165 × 10⁹/L indicates PCAD. (2) If 3.56 < TyG < 4.85: (a) PIV > 368.56 suggests PCAD. (3) If TyG < 4.85 and PIV < 368.56: (a) WBC > 5.62 × 10⁹/L suggests PCAD. The model's diagnostic performance was evaluated, demonstrating an accuracy of 0.88 and an area under the receiver operating characteristic curve (AUC) of 0.86. A schematic of the decision tree is presented in Figure 3.

### 3.5 Feature variable selection via machine learning

Machine learning, bolstered by computational advancements, is widely utilized in medical research for analyzing complex datasets (15–17). Ensemble machine learning models constitute a notable advance, offering robust tools for predicting patient prognosis based on input variables and cohort data. These models substantially alleviate the burden on researchers by automating feature variable selection for prognostic modeling.

**Figure 4:**
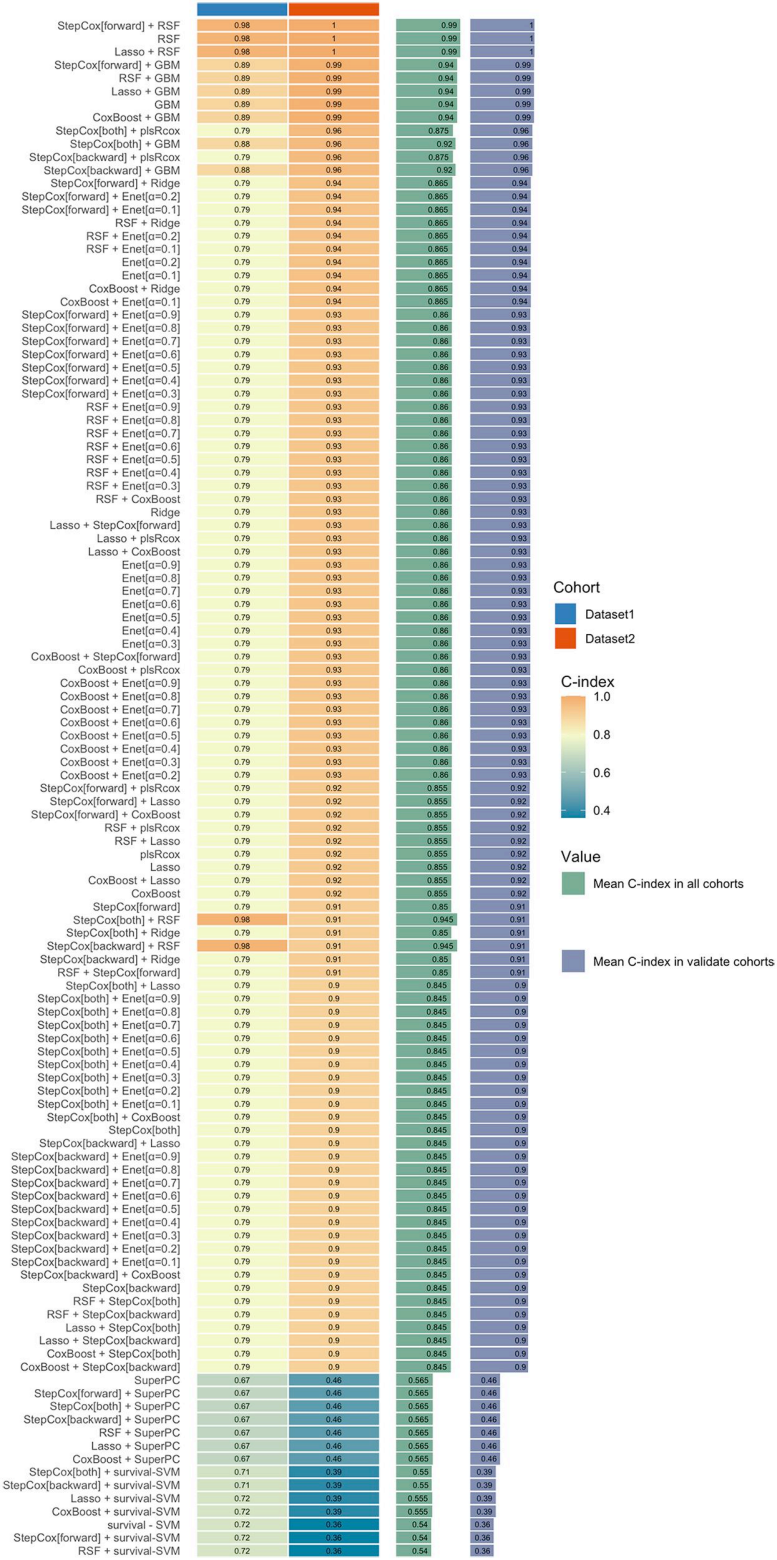
An ensemble model for the prognosis of PCAD. The StepCox[forward]-RSF ensemble model exhibited the highest mean C-index (0.99) across both training and validation cohorts. RSF, random survival forest.

In this study, the Mime1 package in R was used to construct an ensemble model for predicting the prognosis of PCAD. More than 100 predictive models were developed within a k-fold cross-validation framework, with the concordance index (C-index) computed for each model across validation datasets (Figure 4). Among these models, the StepCox [forward]-RSF ensemble model exhibited the highest mean C-index (0.99) across both training and validation cohorts, establishing it as the optimal model.

**Figure 5:**
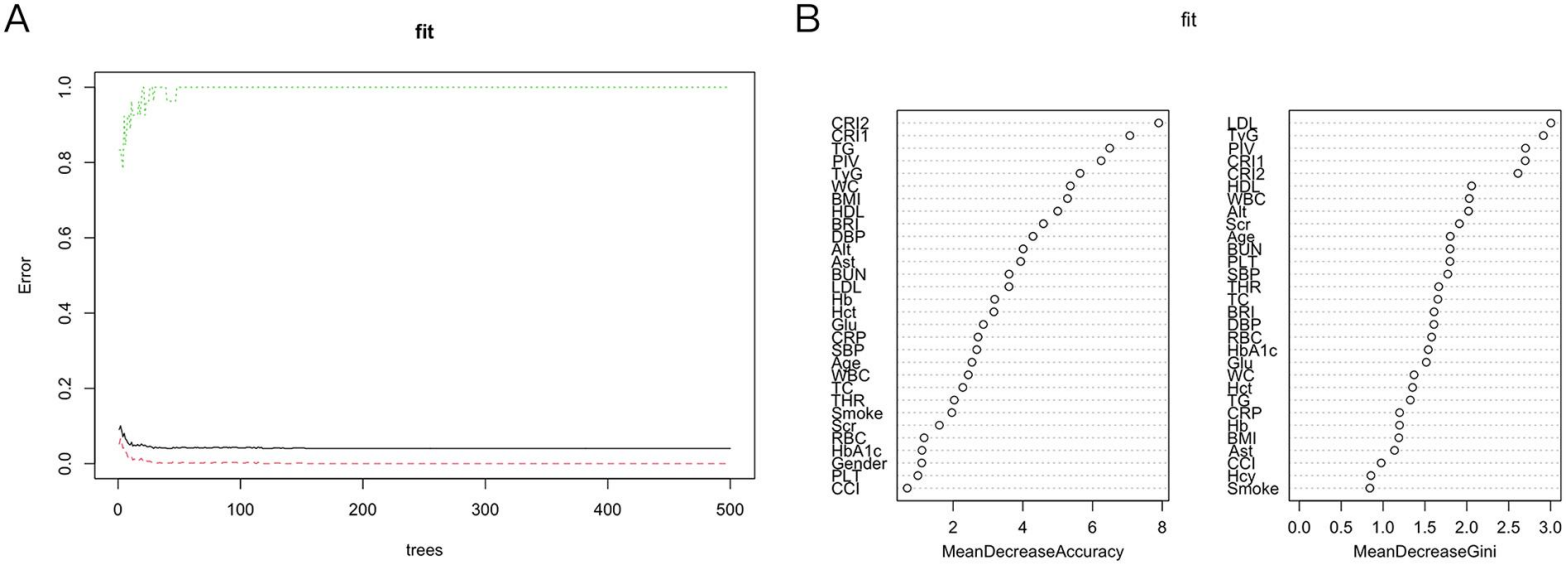
Random survival forest. **(A)** Show that the error rate of the model stabilizes when the number of classification trees exceeds 200. **(B)** RSF: Based on Gini importance evaluation, the five most important features are LDL, TyG, PIV, CRI1, and CRI2. RSF, random survival forest; LDL, low-density lipoprotein cholesterol; TyG, triglyceride-glucose index; PIV, pan-immunoinflammatory value; CRI1and CRI2, castelli risk index 1 and 2.

The 37 feature variables were subsequently evaluated using machine learning methods implemented in R. The Random Survival Forest (RSF) ranked the top five variables as LDL-C, TyG, PIV, CRI-1 and CRI-2, in descending order of importance (Figure 5). Furthermore, the StepCox [forward] method identified 10 key feature variables: PLT, Scr, Hcy, Glu, Smoke, TyG, PIV, CCI, WBC and CRP (Table 3). The consistent identification of TyG and PIV across both methods indicates their strong association with the prognosis of PCAD.

**Table 3:** Univariate regression analysis of PCAD.

Parameter	HR (95%CI)	*P* value	Parameter	HR (95%CI)	*P* value
Age	1.01 (0.97–1.05)	0.59	TG	3.60 (0.61–21.34)	0.15
Gender1	1.24 (0.57–2.70)	0.58	Glu	1.01 (1.01–1.03)	<0.01
WBC	0.78 (0.33–1.86)	0.58	HbA1c	1.24 (0.93–1.65)	0.14
RBC	1.37 (0.46–4.16)	0.56	SBP	1.00 (0.98–1.03)	0.58
Hb	1.98 (0.79–5.00)	0.14	DBP	0.99 (0.96–1.03)	0.67
Hct	0.74 (0.50–1.49)	0.13	BMI	0.98 (0.83–1.16)	0.82
PLT	0.99 (0.98–1.00)	<0.01	WC	1.01 (0.94–1.08)	0.69
Alt	1.00 (0.99–1.00)	0.33	Smoke1	5.5 (2.41–12.82)	<0.01
Ast	1.00 (0.99–1.02)	0.18	Drink1	0.62 (0.24–1.66)	0.35
BUN	0.88 (0.70–1.13)	0.34	CRI1	0.95 (0.34–2.65)	0.92
Scr	0.96 (0.94–0.98)	<0.01	CRI2	1.07 (0.42–2.77)	0.87
Hcy	0.87 (0.77–0.97)	0.01	THR	1.30 (0.41–4.13)	0.64
CRP	0.92 (0.71–1.20)	0.56	TyG	0.08 (0.01–0.71)	0.02
HDL	1.04 (0.11–9.35)	0.96	BRI	0.94 (0.55–1.61)	0.83
LDL	0.97 (0.38–2.50)	0.95	PIV	1.00 (1.00–1.00)	<0.01
TC	1.00 (0.37–2.74)	0.98	CCI	1.21 (1.02–1.44)	0.02

PCAD, premature coronary artery disease; Gender1, gender = men; WBC, white blood cell count; RBC, red blood cell count; Hb, hemoglobin; Hct, hematocrit; PLT, platelets; ALT, alanine aminotransferase; AST, aspartate aminotransferase; BUN, urea nitrogen; Scr, creatinine; Hcy, homocysteine; CRP, c-reactive protein; HDL, high-density lipoprotein cholesterol; LDL, low-density lipoprotein cholesterol; TC, total cholesterol; TG, triglycerides; Glu, blood glucose; HbA1c, glycated hemoglobin; SBP, systolic blood pressure; DBP, diastolic blood pressure; BMI, body mass index; WC, waistline; Smoke1, smoke = yes; Drink1, drink1 = yes; CRI1and CRI2, castelli risk index 1 and 2; THR, triglyceride-to-HDL ratio; TyG, triglyceride-glucose index; BRI, body roundness index; PIV, pan-immunoinflammatory value; CCI, Charleston comorbidity index.

### 3.6 ROC analysis to determine optimal cutoff value

**Figure 6:**
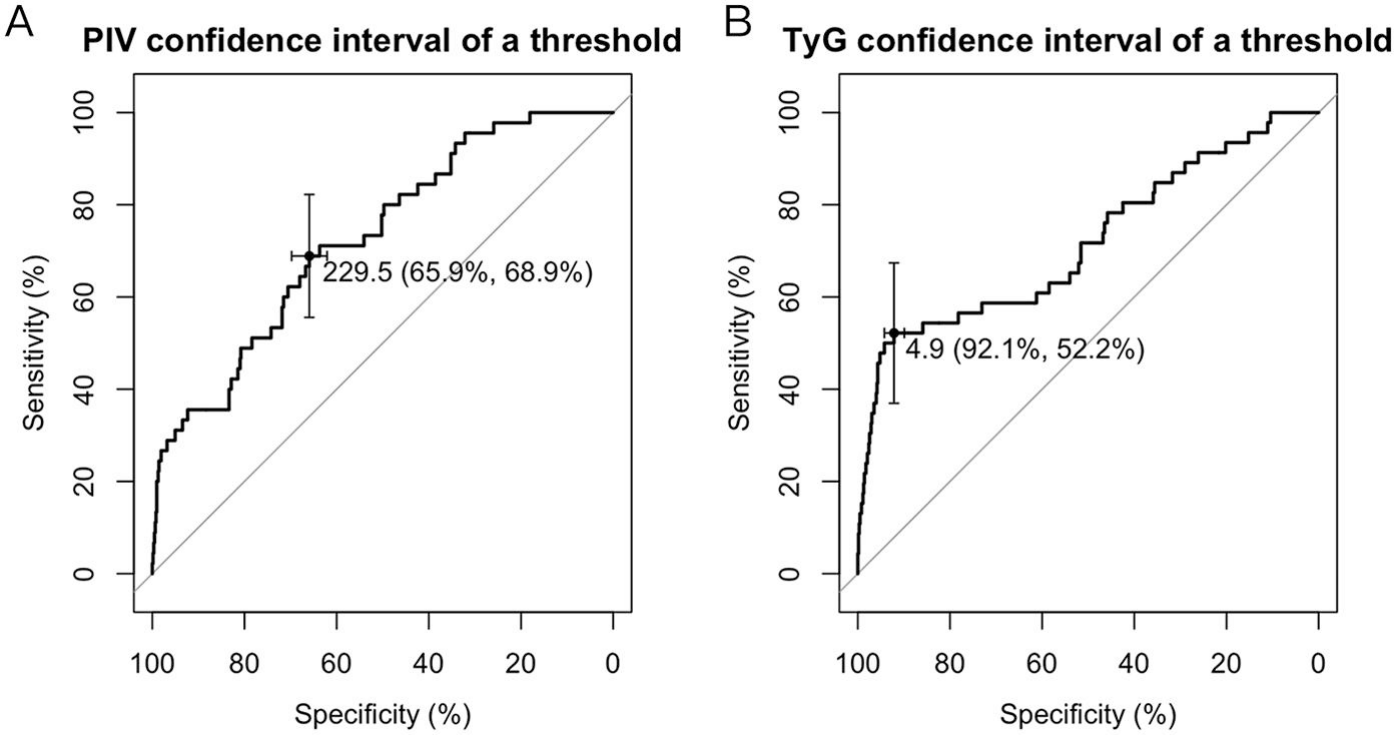
ROC curve. **(A)** The optimal threshold for the PIV index predicted by the PCAD prognostic model was determined to be 229.5, at which the specificity and sensitivity of the PCAD prognostic model were 65.9% and 68.9%. **(B)** The optimal critical value of the TyG index predicts that the PCAD prognostic model is 4.9, at which the specificity and sensitivity of the PCAD prognostic model are 92.1% and 52.2%. TyG, triglyceride-glucose index; PIV, pan-immunoinflammatory value.

ROC curve analysis was conducted to determine optimal cutoff values for the PIV and TyG index, key predictors of prognosis in patients with PCAD. These cut-off values were established to optimize the prognostic model's sensitivity and specificity, delineating thresholds beyond which mortality risk in PCAD patients markedly rises. For PIV, the optimal cutoff value was determined as 229.5. At this threshold, the prognostic model achieved a specificity of 65.9% and a sensitivity of 68.9% (Figure 6A). Likewise, for the TyG index, the optimal cutoff value was established at 4.9, with a specificity of 92.1% and a sensitivity of 52.2% (Figure 6B). These results underscore that PIV and TyG serve as effective biomarkers for stratifying mortality risk in PCAD patients, with the established thresholds balancing diagnostic precision and clinical applicability.

### 3.7 Kaplan–Meier survival analysis

This analysis assessed the impact of varying TyG and PIV levels on the survival outcomes of patients with PCAD over three years. The study population was divided into groups based on cutoff values of 229.5 for PIV and 4.9 for TyG.

**Figure 7:**
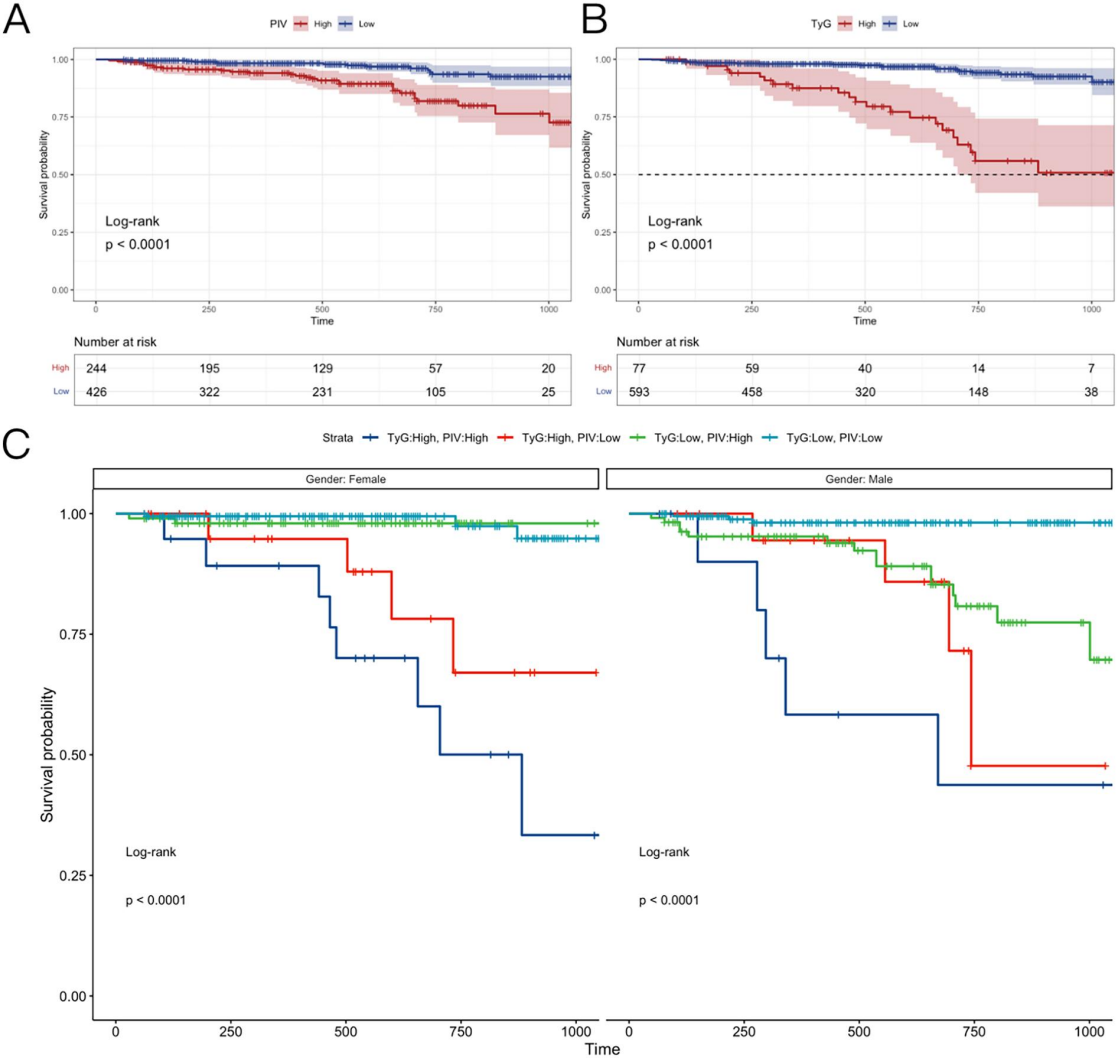
Kaplan–Meier survival analysis. **(A)** The follow-up of patients with different PIV index groups for 36 months. **(B)** The follow-up of patients with different TyG index group for 36 months. **(C)** Men and women were followed up for 36 months in different PIV + TyG groups. TyG, triglyceride-glucose index; PIV, pan-immunoinflammatory value.

Analysis by PIV Levels: The population was categorized into two groups according to the PIV threshold of 229.5: (1) Low PIV group (PIV < 229.5); (2) High PIV group (PIV ≥ 229.5). Kaplan–Meier survival curves (Figure 7A) demonstrated a markedly lower survival rate in the high PIV group compared to the low PIV group over three years. This indicates that elevated PIV levels correlate with a poorer prognosis in PCAD patients.

Analysis by TyG Levels: Likewise, the population was divided into two groups based on the TyG threshold of 4.9: (1) Low TyG group (TyG < 4.9); (2) High TyG group (TyG ≥ 4.9). Figure 7B shows that the high TyG group exhibited a lower survival rate than the low TyG group, suggesting that elevated TyG levels are linked to heightened mortality risk in PCAD.

Combined effects of PIV and TyG: The combined influence of PIV and TyG levels on survival rates was examined, with outcomes stratified by gender (Figure 7C). Four groups were evaluated: (1) High PIV + High TyG: This group displayed the lowest survival rates across genders, highlighting the heightened risk associated with elevated levels of both indices. (2) Low PIV + Low TyG: Conversely, this group exhibited the highest survival rates, indicating a protective effect when both indices fall below their thresholds. (3) High PIV + Low TyG and (4) Low PIV + High TyG: The high PIV + low TyG group demonstrated a higher survival rate than the low PIV + high TyG group. This suggests that the TyG index is a stronger prognostic indicator in PCAD, with lower TyG levels mitigating the adverse effects of elevated PIV.

Gender-Specific Survival Trends: In the low PIV + low TyG group, modest gender differences emerged: (1) Females exhibited higher survival rates than males in the early three-year period; (2) Subsequently, female survival rates decreased, falling below male rates in later stages. The reasons for this gender-specific trend are addressed in the discussion section.

Kaplan–Meier survival analysis revealed that elevated PIV and TyG levels correlate with reduced survival in PCAD patients. The high PIV + high TyG combination yielded the poorest outcomes, whereas the low PIV + low TyG combination was associated with the most favorable prognosis. Notably, the TyG index exerts a greater influence on survival, as demonstrated by the superior outcomes in the high PIV + low TyG group compared to the low PIV + high TyG group. These results, alongside gender-specific patterns, provide critical insights for risk stratification and prognostic evaluation in PCAD.

## 4 Discussion

The prevalence of PCAD has risen in recent years, with a worse prognosis than CAD in older adults. In contrast to CAD in older populations, PCAD is characterized by fewer conventional risk factors yet manifests more acutely, often as an initial acute coronary syndrome in most patients. This acute onset elevates mortality rates among affected individuals. Moreover, the prolonged life expectancy of younger patients increases the likelihood of long-term complications, including recurrent cardiovascular events or heart failure. Thus, identifying reliable prognostic indicators, implementing effective prevention strategies, and developing targeted early interventions are critical to improving survival rates and quality of life for PCAD patients.

This study employs diverse machine learning algorithms to examine risk factors linked to PCAD and their prognostic impact, providing a foundation for early detection and primary prevention. A predictive model was constructed using various machine learning algorithms to forecast both the diagnosis and prognosis of PCAD. The diagnostic model attained an accuracy of 0.88, indicating robust performance in identifying PCAD. Furthermore, the PIV and TyG index emerged as key prognostic indicators for PCAD. Receiver operating characteristic (ROC) curve analysis established optimal cutoff values for these indices (PIV > 229.5, TyG > 4.9) and evaluated their sensitivity and specificity for prognostic stratification in PCAD. Survival analysis confirmed that elevated PIV and TyG levels correlate with a poorer prognosis, with the TyG index exhibiting a notably strong association.

Extensive research has established atherosclerosis as a low-grade, non-infectious inflammatory condition (18–21). This condition is characterized by inflammation-induced endothelial dysfunction, initiating key processes: (1) Lipoprotein accumulation beneath the impaired endothelium; (2) Leukocyte recruitment to the inflammatory site; (3) Increased vascular permeability, worsening the condition. Recruited monocytes differentiate into macrophages, adopting pro-inflammatory or anti-inflammatory phenotypes based on the local microenvironment. The balance between these phenotypes critically governs atherosclerosis progression or resolution (22).

Systemic and localized inflammation, affecting the entire body and specific vessels respectively, are pivotal in the development and progression of cardiovascular disease. Thus, early and precise detection of inflammation, combined with timely intervention, is crucial for enhancing outcomes in patients with PCAD.

Although single biomarkers provide limited predictive value for cardiovascular risk assessment, recent focus has shifted to multi-biomarker panels. These biomarker combinations demonstrate substantial potential for improving the precision of cardiovascular disease outcome predictions.

The PIV is a systemic inflammation biomarker derived from specific blood cell subgroup counts. It combines neutrophil, monocyte, lymphocyte, and platelet counts—cell types integral to inflammation—to yield a composite measure of immune-inflammatory status (23–25). PIV is increasingly valued for its prognostic utility across conditions such as cancers, cardiovascular diseases, and metabolic syndrome (15, 16, 26). Given the established inflammation-CAD link, PIV's ability to predict CAD severity and complications has attracted considerable interest. Ayşe Irem Demirtola *et al*. (17) reported that elevated PIV levels correlate with increased atherosclerotic lesion severity in CAD patients. Likewise, Bektas Murat et al. (20) noted that higher PIV levels correlate with elevated long-term mortality in ST-elevation myocardial infarction (STEMI) patients, although no significant association emerged with in-hospital mortality. These results support the notion that systemic inflammation drives CAD progression. Moreover, PIV outperforms other inflammatory indices—such as the systemic immune-inflammation index (SII), platelet-to-lymphocyte ratio (PLR), and neutrophil-to-lymphocyte ratio (NLR)—in predicting post-percutaneous coronary intervention (PCI) prognosis and coronary artery stenosis extent in STEMI patients (21).

Despite these insights, PIV's role in PCAD, a CAD subset in younger individuals, remains largely unexamined. Current studies have not fully explored the PIV-PCAD relationship, underscoring a significant literature gap. Given PCAD's distinct profile—fewer conventional risk factors yet a more acute course—examining PIV's predictive utility in this population could provide critical prognostic and therapeutic insights.

The TyG index, derived from plasma triglycerides and fasting glucose levels, offers a simple, non-invasive method to assess insulin resistance (IR). It is widely applied to evaluate IR, predict metabolic dysregulation, and assess cardiovascular disease (CVD) risk. Hyperglycemia and hypertriglyceridemia are established drivers of CVD, contributing to endothelial dysfunction, inflammation, and atherosclerosis—central mechanisms in cardiovascular complications. In healthy individuals, insulin regulates glucose and lipid metabolism by facilitating glucose uptake, promoting glycolysis, and maintaining lipid homeostasis. However, in IR, these processes falter, leading to impaired glucose uptake, reduced glycolysis, and dyslipidemia, which are closely tied to adverse cardiovascular outcomes (27). The TyG index provides a practical alternative to traditional IR measures like the hyper insulinemic-euglycemic clamp or HOMA-IR (28). Studies consistently link a higher TyG index to increased risks of myocardial infarction, stroke, and heart failure, even after adjusting for conventional risk factors such as age, smoking, and hypertension. Its integration of triglycerides and glucose enhances its robustness in predicting CVD risk, particularly in metabolic syndrome, where it often outperforms other IR markers.

Despite its established role in CVD (29–32), the TyG index's association with PCAD remains underexplored. Given PCAD's unique metabolic and inflammatory profile, investigating the TyG index in this context could refine risk stratification and guide early interventions.

The advent of machine learning (ML) has transformed vast datasets into actionable models, significantly enhancing diagnostic precision. While ML models for cardiovascular disease likelihood and prognosis are emerging (33, 34), many are limited by small sample sizes or a focus on older populations, with few large-scale studies addressing PCAD. Our study bridges this gap by developing a diagnosis and prognosis model tailored to PCAD patients. We employed multiple ML methods with cross-validation to select key variables, diverging from traditional training-validation splits. Our findings confirm a significant association between PIV, the TyG index, and PCAD occurrence, with elevated levels linked to poorer prognosis, reinforcing inflammation's foundational role in atherosclerosis. Notably, in the low PIV + low TyG cohort, women exhibited lower survival rates than men over time, possibly due to post-menopausal estrogen decline reducing atherosclerosis resistance.

## 5 Conclusions

The combined evaluation of PIV, TyG, and WBC offers robust diagnostic and prognostic value for PCAD, with elevated PIV and TyG levels indicating a poor prognosis, underscoring their potential as clinical biomarkers.

## 6 Limitations

Despite these advances, limitations persist. A limitation of our study is that our definition of PCAD was based on angiographic stenosis ≥50%, which may not capture patients with early subclinical atherosclerosis. Future studies should aim to establish a consensus definition to improve comparability. This study was conducted based on data from a single center, which may limit the generalizability of our findings and raises the possibility of overfitting in the predictive model. Although the internal performance was robust, external validation using multicenter or publicly available datasets is warranted to confirm the reliability and broader applicability of the model in different populations and clinical settings. To enhance clinical applicability, it is worth noting that the components of PIV and TyG are typically part of routine laboratory tests, available within a short time frame in most emergency or outpatient settings. Therefore, their integration into clinical decision-making pathways is feasible in many healthcare systems. Nonetheless, in resource-limited environments or when immediate lab access is not possible, simplified models based on clinical features or point-of-care tests may be explored, though potentially at the expense of diagnostic accuracy. Further studies are needed to validate such surrogate models. Minor CommentsSingle-time-point measurements of glucose and lipid levels overlook temporal variations, and the retrospective design may introduce confounding and bias. Futhermore, the follow-up information in this retrospective study was restricted to all-cause mortality, and detailed coronary events such as PCI, re-PCI, or myocardial infarction were not uniformly available. In addition, data on peripheral atherosclerosis and related vascular events were lacking. These factors may have limited our ability to comprehensively evaluate cardiovascular outcomes. Future prospective multicenter studies with standardized collection of interventional and peripheral vascular events are needed to validate and extend our findings. Future research should leverage larger, multicenter cohorts, extended follow-ups, and randomized controlled trials to enhance predictive accuracy and validate these findings.

## Data Availability

The data supporting the findings of this study are available from the corresponding author upon reasonable request.
